# 

**Published:** 1842-07

**Authors:** 


					""
THE
BRITISH AND FORE
MEDICAL REVIEW
OR
QUARTERLY JOURNAL
PRACTICAL MEDICINE AND SURGERY
LIBRARY OF THE
Orleans Fariuh 11 u'.i; ?
EDITED BY
JOHN FORBES M.D. F.R.S. F.G.S.
VOL. XIV.
JULY?OCTOBER 1842.
LONDON
JOHN CHURCHILL, PRINCES STREET, SOHO.
MRCCCXLJI.
Printed by C. Adlard, Bartholomew Close.

				

